# Worldwide Glycoscience Informatics Infrastructure: The GlySpace Alliance

**DOI:** 10.1021/jacsau.2c00477

**Published:** 2022-12-02

**Authors:** Frederique Lisacek, Michael Tiemeyer, Raja Mazumder, Kiyoko F. Aoki-Kinoshita

**Affiliations:** † Proteome Informatics Group, SIB Swiss Institute of Bioinformatics, University of Geneva, Geneva CH-1227, Switzerland; ‡ Complex Carbohydrate Research Center, University of Georgia, Athens, Georgia 30602, United States; § George Washington University, Washington, District of Columbia 20037, United States; ∥ Glycan and Life Systems Integration Center (GaLSIC), Soka University, Hachioji, Tokyo 192-8577, Japan; △ Computer Science Department & Section of Biology, University of Geneva, Geneva CH-1227, Switzerland

**Keywords:** databases, web portals, glycoscience, carbohydrates, bioinformatics

## Abstract

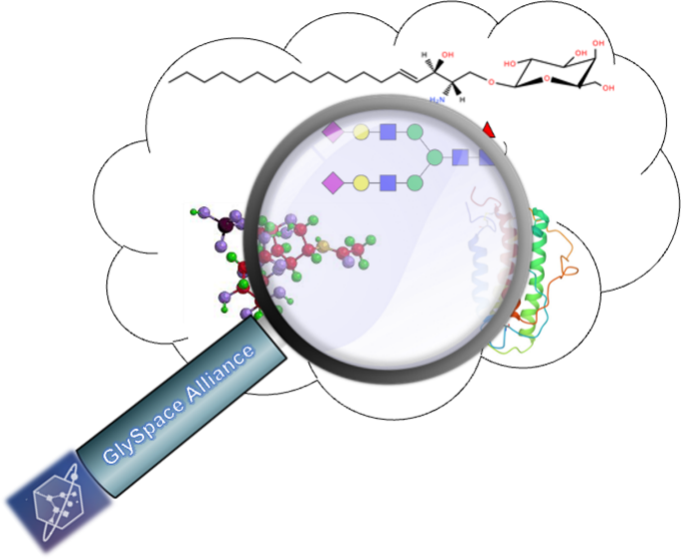

The GlySpace Alliance was formed in 2018 among the principal investigators of three major glycoscience portals: Glyco@Expasy, GlyCosmos, and GlyGen, representing Europe, Asia, and the United States, respectively. While each of these portals has its unique user interface, the aim is to provide the same basic data set of glycan-related omics data. These portals will be introduced with the aim to enable users to find their target information in the most efficient manner, in particular, in terms of the chemical structures of glycans and their functions.

## Introduction

### History of Glycan Databases

The first carbohydrate repository was developed by the Complex Carbohydrate Research Center (CCRC) at the University of Georgia. It was called the Complex Carbohydrate Structure Database (CCSD) but later known as CarbBank, after the name of the software used to access CCSD.^1^ The database contained over 40 000 entries upon the end of its development in the late 1990s. The data was made publicly available, however, and taken up by three major databases in the United States, Europe, and Japan: the Consortium for Functional Glycomics (CFG),^2^ Glycosciences.de,^3^ and KEGG GLYCAN,^4^ respectively. While the CFG has now been incorporated into the National Center for Functional Glycomics (NCFG), the data is still available on the original web site. Both Glycosciences.de and KEGG GLYCAN continue to update and make available the glycan data from CarbBank, integrating them with their respective resources. Glycosciences.de focuses on the 3D structures of glycans, extracting carbohydrates from the Protein Data Bank (PDB), while KEGG GLYCAN continues to populate their PATHWAY information with glycan-related genes and diseases.

Many other databases on carbohydrates have also been developed in the meantime. For example, the Bacterial Carbohydrate Structure Database (BCSDB) has combined with their plant and fungal carbohydrate structure database to form the Carbohydrate Structure Database (CSDB).^5^ The well-known CAZy database of carbohydrate active enzymes also continues to develop with new glycogene families being added continuously.^6^

### Glycan-Centered Web Portals: The GlySpace Alliance

On the other hand, new web portals developed specifically for integrating omics data centered on glycans have also emerged. Glycomics@ExPASy,^7^ recently renamed Glyco@Expasy, is housed at the well-known Expasy Swiss Bioinformatics Resource Portal and provides user-friendly access to various databases and software tools. GlyCosmos^8^ was developed in Japan under their database integration program to integrate glycan data across all life science domains using Semantic Web technologies. GlyGen^9^ was developed under the U.S. NIH Common Fund program to integrate and disseminate carbohydrate and glycoconjugate data. Because of the overlap in these portals, these three projects have agreed to communicate and collaborate as the GlySpace Alliance to ensure the integrity of glycan-based data around the world.

## Nomenclature

While comprehensive reviews of various textual and graphical representations of glycans are already available, here we describe the three major representations used across the portals of the GlySpace Alliance.^10^

### SNFG

**Figure 1:**
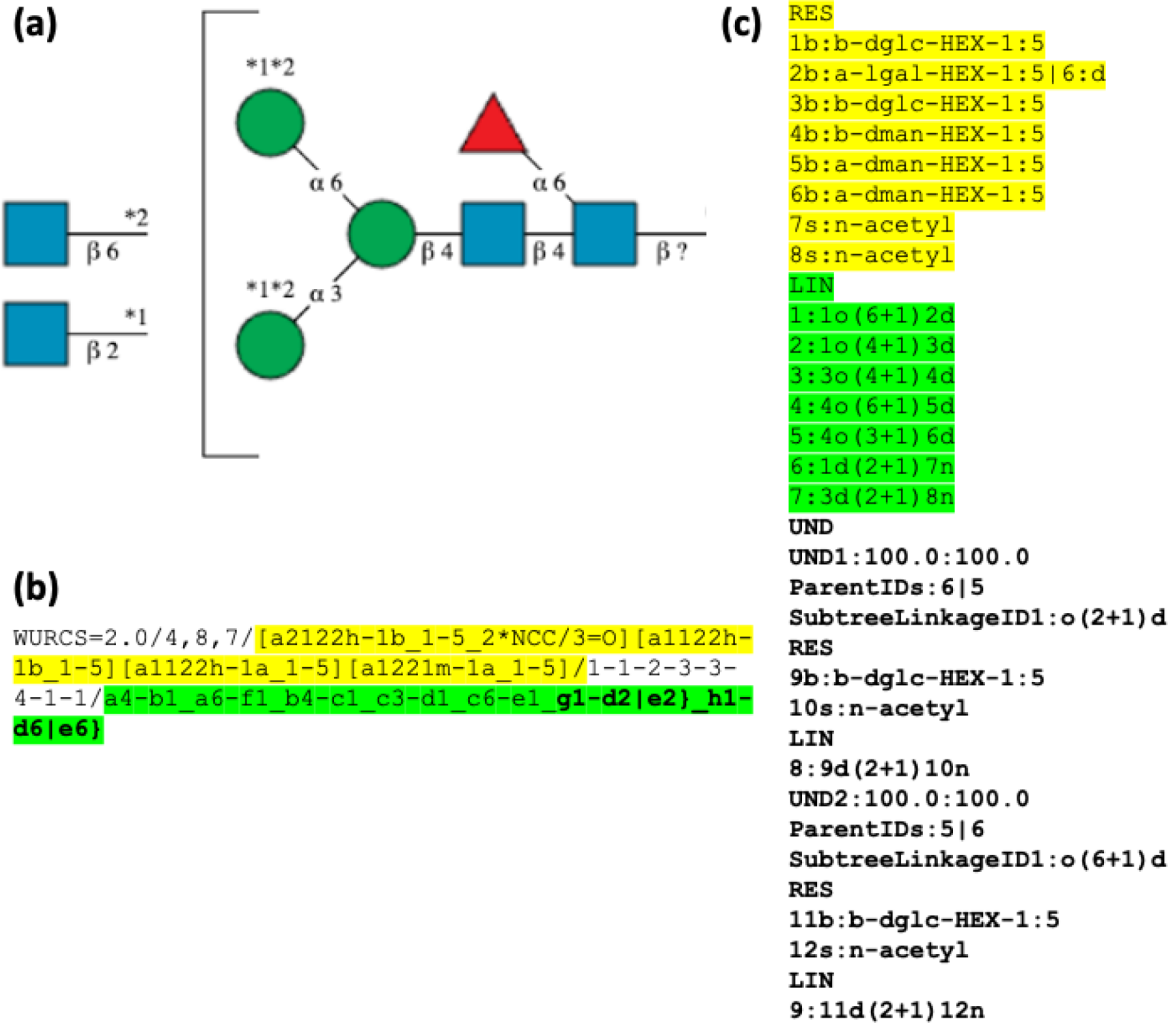
Example of an N-linked glycan with various possible attachments of monosaccharides. (a) SNFG representation, drawn using SugarDrawer, where the symbols indicate the following: blue square = *N*-acetylglucosamine, green circle = mannose, red triangle = fucose. (b) WURCS representation, whereas (c) GlycoCT representation for the same structure. Yellow highlighted text represents the monosaccharide residues, green highlighted text represents the linkage information, and bold text represents the ambiguous linkages.

The Symbol Nomenclature For Glycans (SNFG)^11^ is the proposed standard for representing glycan structures graphically. Specific symbols are proposed for specific monosaccharides with defined RGB codes for coloring which can be distinguished even if printed in monochrome. The SNFG glycan pages at https://www.ncbi.nlm.nih.gov/glycans/snfg.html give examples of usage, including how glycans should be depicted with substituents and ambiguous linkages. Figure 1 illustrates a complex example of an N-linked glycan with possible attachment of three monosaccharides at various positions.

### WURCS

WURCS (version 2)^12^ is the text representation of glycans used in GlyTouCan.^13^ It is a linear string format designed to uniquely represent any glycan composition, structure, or fragment. Rules are devised such that when the WURCS representation of a glycan is normalized, the same unique representation is obtained, ensuring that any glycan structure can be represented uniquely using the official WURCS string. Several tools for drawing and converting glycans into WURCS format are available, as listed in Table 1. The latest information can be obtained from the WURCS working group homepage at https://www.wurcs-wg.org/.

**Table 1:** List of Software Tools for Obtaining WURCS Formatted Strings for Glycans

tool name	description	URL
GlycanFormat Converter Web	various online tools to convert from glycan text formats such as GlycoCT and IUPAC into WURCS are available	https://glyconavi.org/Tools/tool/gfc.php
GlyCosmos Web API	user interface for various APIs provided by GlyCosmos, including conversion of WURCS to images in SNFG format	https://api.glycosmos.org/
Glycan converters	various glycan text format converters used by GlyCosmos, including tools to normalize WURCS, obtain image files, as well as GlyTouCan IDs	https://glycosmos.org/glycans/converter
SugarDrawer and GlycanBuilder2	graphic user interfaces at GlyCosmos to search glycans by drawing them on a canvas and querying glycan databases such as GlyTouCan	https://glycosmos.org/glycans/graphic

## GlycoCT

GlycoCT is the oldest and currently most often used format for representing glycans.^14^ It is used mainly in Glyco@Expasy and software tools such as GlycanBuilder and GlycoWorkBench,^15^ which are tools used for analyzing glycomics mass spectra data. GlycoCT is a multiline format for representing glycan structures and compositions. The format is intended to be human readable and easily compressed and includes a canonicalization algorithm to ensure that there is only a single representation for a glycan structure. Software libraries such as glypy^16^ for Python users are available, and many of the software tools in Table 1 also support export into GlycoCT format.

## Glyco@Expasy

Glyco@Expasy centralizes references to strictly web-based glycoinformatics resources, whether hosted or not on Expasy. The aim of this portal is to make glycobiology more accessible to scientists in both the glycosciences and the protein sciences. It strongly advocates for presenting glycans as mediators of protein–protein interactions. The major sections of Glyco@Expasy will be described next.

### Main Portal

**Figure 2:**
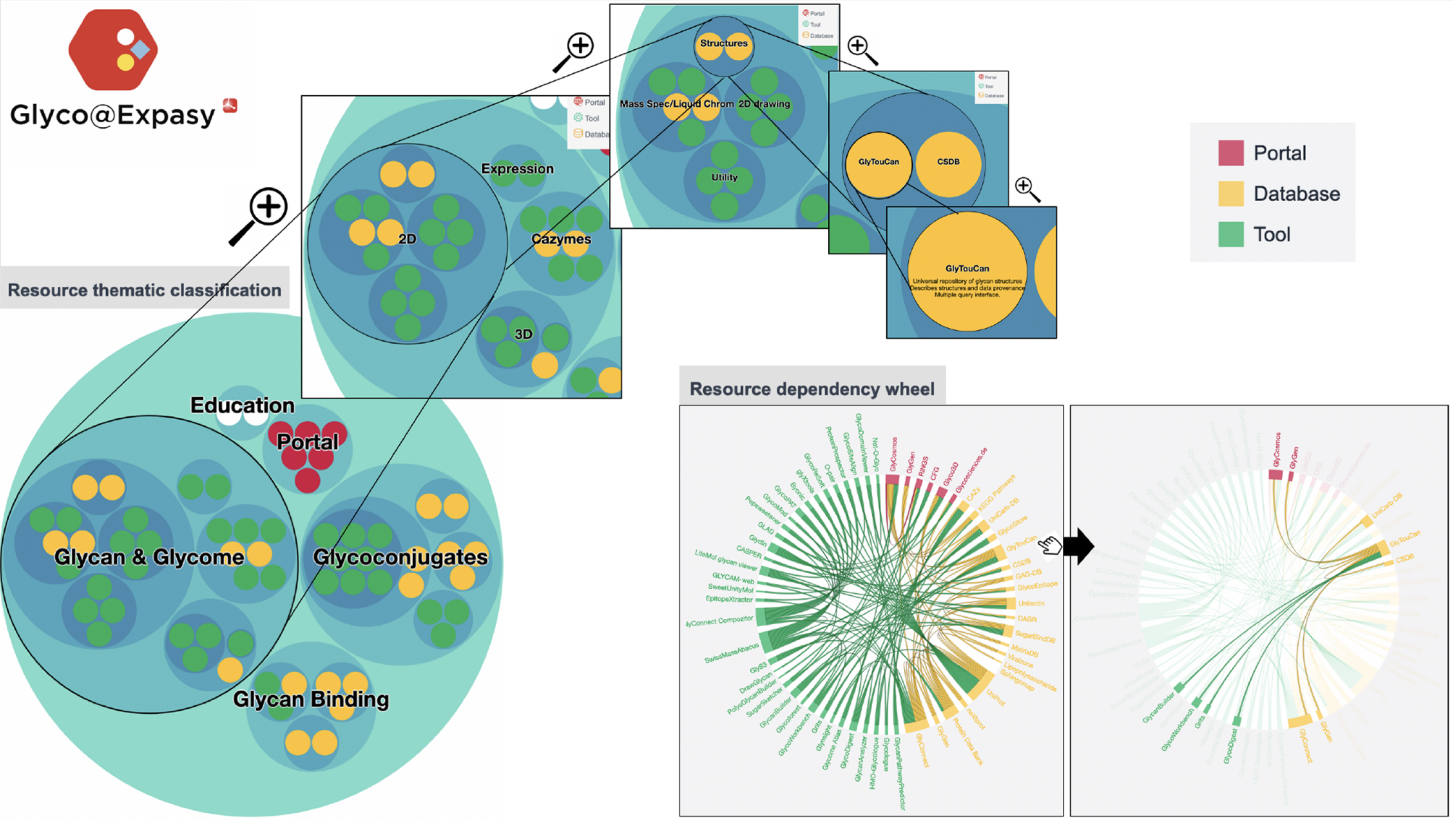
Screenshots on the left featuring successively zoomed in information (zoom enabled by mouse click on bubble) ending on the GlyTouCan database as an example of use of the Glyco@Expasy thematic classification. Screenshots on the bottom right show the effect of mousing over the dependency wheel with the example of GlyTouCan. Color code for resources is shown in the upper right corner.

The landing page displays a series of clickable, colored circles (bubbles) representing web portals (red), tools (green), and databases (yellow) in the glycoscience domains (Figure 2). To help users select an appropriate database or software tool, the collected resources are categorized into a hierarchy representing the different fields of glycobiology, such as glycans, glycoconjugates, glycan binding, etc. The checkbox for each term triggers zooming in the bubble chart to focus on the theme of interest. Users can zoom out by clicking outside the circle in focus. Zooming in is also possible by clicking on a circle of interest. All zooming operations systematically check or uncheck boxes in the hierarchy of terms on the left. Information regarding the resource(s) in a selected circle is displayed in the middle of the page, along with a short description, a link to the resource, and a link to the related publication. A second tab displays an interactive dependency wheel with the same color scheme to highlight relationships between resources. Glyco@Expasy strives to update information at least yearly, as new web-based glycoinformatics resources are being released or old ones outdated.

## Glycans and Glycomes

This section includes carbohydrate enzymes (CAZymes), gene expression, and glycan structures in 2D and 3D forms.

### CAZymes

At the time of this writing, the CAZyme section includes the CAZy and KEGG Pathway databases as well as several tools that make use of such glycogene information, such as to predict glycan structures or to digest glycans.

### Two-Dimensional Structures

The section on 2D structures contains several subgroups based on interest: mass spectrometry/liquid chromatography (MS/LC), structure databases, 2D drawing tools, and utility tools. MS/LC includes the databases UniCarb-DB^17^ and GlycoStore,^18^ which are databases known to store such information for glycans, and tools such as Glycoforest,^19^ GlycoWorkbench, and GRITS.^20^ Utility tools itemize in-house integrative tools that ease navigation across resources, such as substructure search.^21^

### Three-Dimensional Structures

The 3D structure section focuses on the spatial conformations of glycans and includes two subsections: 3D viewers/predictors and NMR. The former further contains links to tools for predicting and displaying glycans in 3D, including SweetUnityMol,^22^ GLYCAN-web, and LiteMol viewer.^23^ Under NMR is the CASPER tool for assigning chemical shifts to NMR data.^24^ The GAG-DB is also available under this section as a resource for accessing the 3D structures of glycosaminoglycans.^25^

### Glycan Binding

The glycan binding section consists of three subsections: glycan epitopes, glycan-binding proteins, and glycan–protein interactions. Glycan epitopes include the GlycoEpitope database^26^ and two tools: Glydin^27^ and GLAD.^28^ Glydin is an online tool for investigating various known carbohydrate epitopes accumulated from various sources including the literature and the GlycoEpitope database. GLAD is an online tool to investigate glycan microarray experiments and the glycan binding epitopes that can be obtained as a result.

The glycan-binding proteins subsection consists of two databases: the Database of Anti-Glycan Reagents (DAGR)^29^ and UniLectin,^30^ a curated and predicted lectin database based on a classification of protein folds. On the other hand, the glycan–protein interactions section consists of three databases: MatrixDB,^31^ SugarBindDB,^32^ and ViralZone.^33^ MatrixDB is a database of interactions in the extracellular matrix, much of which involves proteoglycans. SugarBindDB is a database of glycan and pathogen interactions, whereas ViralZone is a database of virus information in general, provided by Expasy.

### Glycoconjugates

In Glyco@Expasy, the glycoconjugates section is divided into glycolipids, proteins, and proteome, glycoproteins (including intact glycopeptides), and glycosites. Without going into too much detail, the glycolipids, protein, and proteome sections consist of databases, whereas intact glycopeptides and glycosites include various tools for analyzing such data. Other than intact glycopeptides, glycoproteins also include two databases GlyGen and GlyConnect,^34^ the latter of which is the main curated glycoprotein database that is shared across the GlySpace Alliance.

## GlyCosmos

GlyCosmos is the official portal of the Japanese Society for Carbohydrate Research (JSCR), but its scope is focused on integrating omics data centered on glycans at a worldwide scale. Semantic Web technologies are used for integration and supplementation of semantics to the data directly, such that not only users but also researchers can learn and glean new knowledge from the data integrated across domains. At the time of this writing, version 3.0 provides four sections: repositories, data resources, tools, and standards.

### Repositories

The repositories section lists the various data repositories whereby users can submit their data. This includes glycan structures for GlyTouCan,^13^ glycomics mass spectrometry data for GlycoPOST^35^ (raw data) and UniCarb-DR^36^ (glycan structures), and GlyComb for glycoconjugate data (currently only glycopeptides).

### Data Resources

The data resources section is organized based on the type of data being targeted: glycogenes, glycoproteins, lectins, glycolipids, glycans, pathways, diseases, and organisms. Each data resource list has a header indicating from what databases/resources the data has been integrated and the date when it was obtained. Filtering options are available on the left, where the different columns to be displayed can be selected and keywords can be entered to search for specific terms in different fields. AND and OR search can be chosen to either search for entries that satisfy all criteria or any of the entered criteria, respectively. The headers of the table in the center panel can be clicked to sort in decreasing or increasing order. Here, we describe the content that is common across the GlySpace Alliance, namely, the Data Resources on glycogenes, glycoproteins, and glycans will be described.

### Glycogenes

The glycogenes section is an integrated list of glycan-related genes, including glycosyltransferases and hydrolases, accumulated from KEGG BRITE,^37^ the Glycogene Database (GGDB),^38^ and others. Each entry has its own detailed page identified by gene ID. This page contains the translated proteins, details about the KEGG BRITE annotations, details about any reactions in which it is involved, known diseases, and links to ChIP-Atlas^39^ and LIPID MAPS,^40^ where available.

### Glycoproteins

The glycoproteins section lists the glycoproteins accumulated from UniProt,^41^ GlyConnect,^34^ and GlyGen.^9^ Each entry detail page has several sections listed in the contents on the upper right: a summary, annotations from UniProt, sequence information highlighting the *N*-glycosylation sequon, a viewer to browse the glycosylation features of the sequence, related pathways, expression information displayed graphically based on the Human Protein Atlas,^42^ disease information, 3D structures from the Protein Data Bank (PDB),^43^ and references from the literature. If the entry is a lectin (glycan-binding protein) then lectin information is also displayed, for example, from UniLectin,^30^ which provides 3D data, the Lectin frontier Database (LfDB),^44^ from which the top three binding glycans based on frontal affinity chromatography are listed, and MCAW-DB,^45^ which displays the glycans that are recognized by the given lectin as a glycan profile.

### Glycans

Glycan data are automatically retrieved from GlyTouCan on a weekly basis. They are then supplemented with additional metainformation such as IUPAC Condensed^46^ and GlycoCT representations, monoisotopic mass, and subsumption level, indicating its level of structural detail. The entry detail page additionally displays the 3D atomic structure of the glycan, where possible (all atomic information needs to be known), along with external links to other relevant databases. If the glycan is annotated in GGDB as a substrate or product of one of its genes, the corresponding data is displayed on this page. Similarly, if it is a glycan that has been analyzed using LC/MS in GALAXY, a list of the degrading enzymes and other similar glycans in GALAXY are displayed. Core protein information from GlyGen and GlycoProtDB^47^ as well as epitope information from GlycoEpitope^26^ and LfDB and tissue expression as annotated by GlycomeAtlas^48^ are also listed.

## Tools

Many users have needs to more easily analyze glycan-related information, and the GlySpace Alliance member portals provide various tools to aid users to do so. In GlyCosmos, GlycoMaple^49^ and the drawing tools SugarDrawer^50^ and GlycanBuilder in addition to the various glycan format conversion tools described in the Nomenclature section are available.

### GlycoMaple

**Figure 3:**
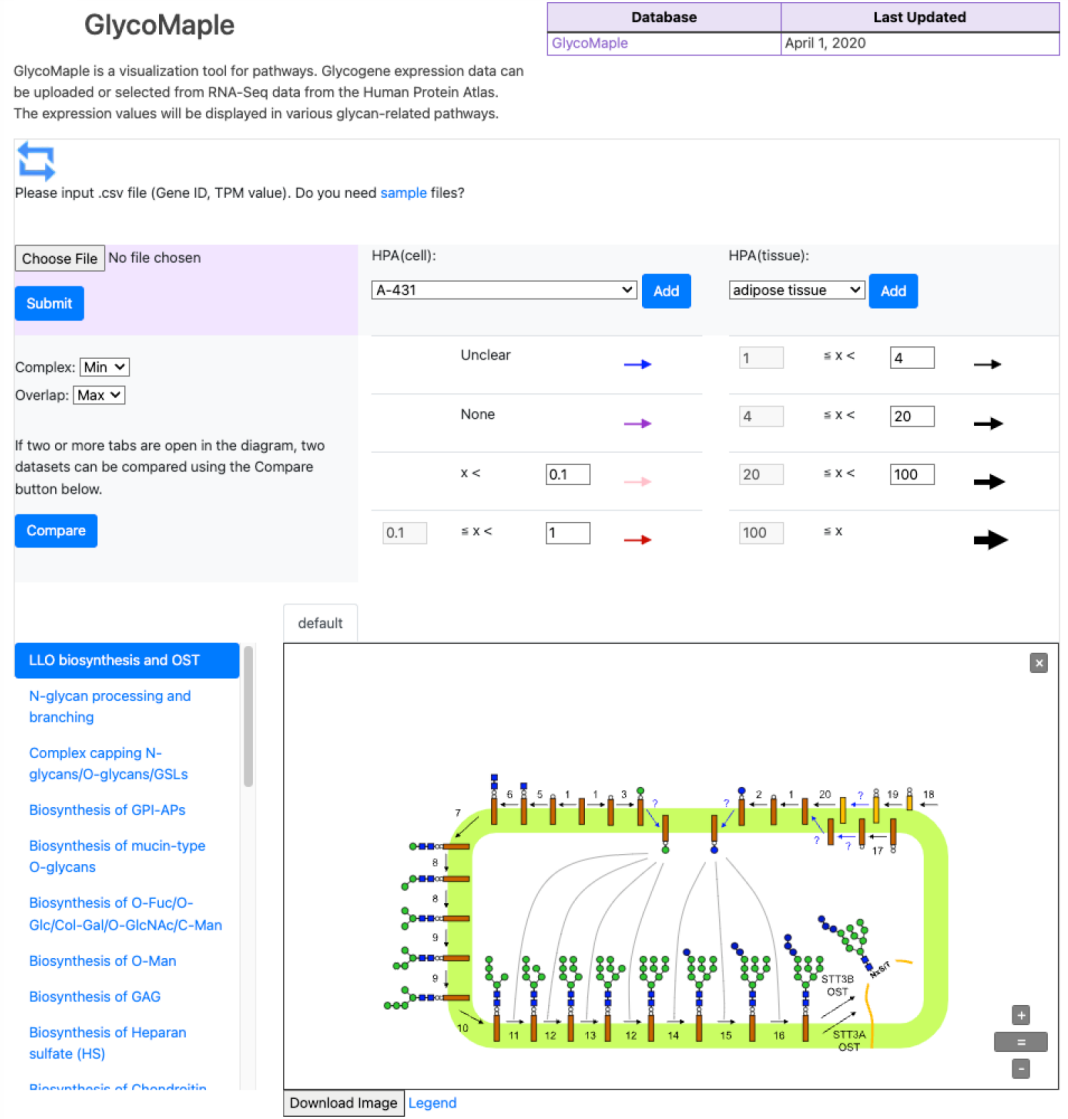
Screenshot of the GlycoMaple tool, which allows users to analyze glycogene expression on top of the various glycan biosynthesis pathways known.

GlycoMaple is a web tool to aid users in understanding the complex relationships between glycogenes and glycans. All of the major biosynthetic pathways of various glycans are provided, as shown in Figure 3. Gene expression data from the Human Protein Atlas can be selected from the HPA(cell) and HPA(tissue) pull-down menus to select from cell lines and tissues, respectively. Users can also upload their own data by choosing their file in the upper left. The expression values will be displayed according the expression value ranges selected in the panel above the pathway diagram. All of the pathways will be updated to reflect the expression data, which allows users to assess the relationships between various glycan types affected by the same gene expression data.

### Standards

The standards section simply provides links to the standard ontologies and nomenclature used in GlyCosmos. These were mainly described earlier in the Nomenclature section.

## GlyGen

**Figure 4:**
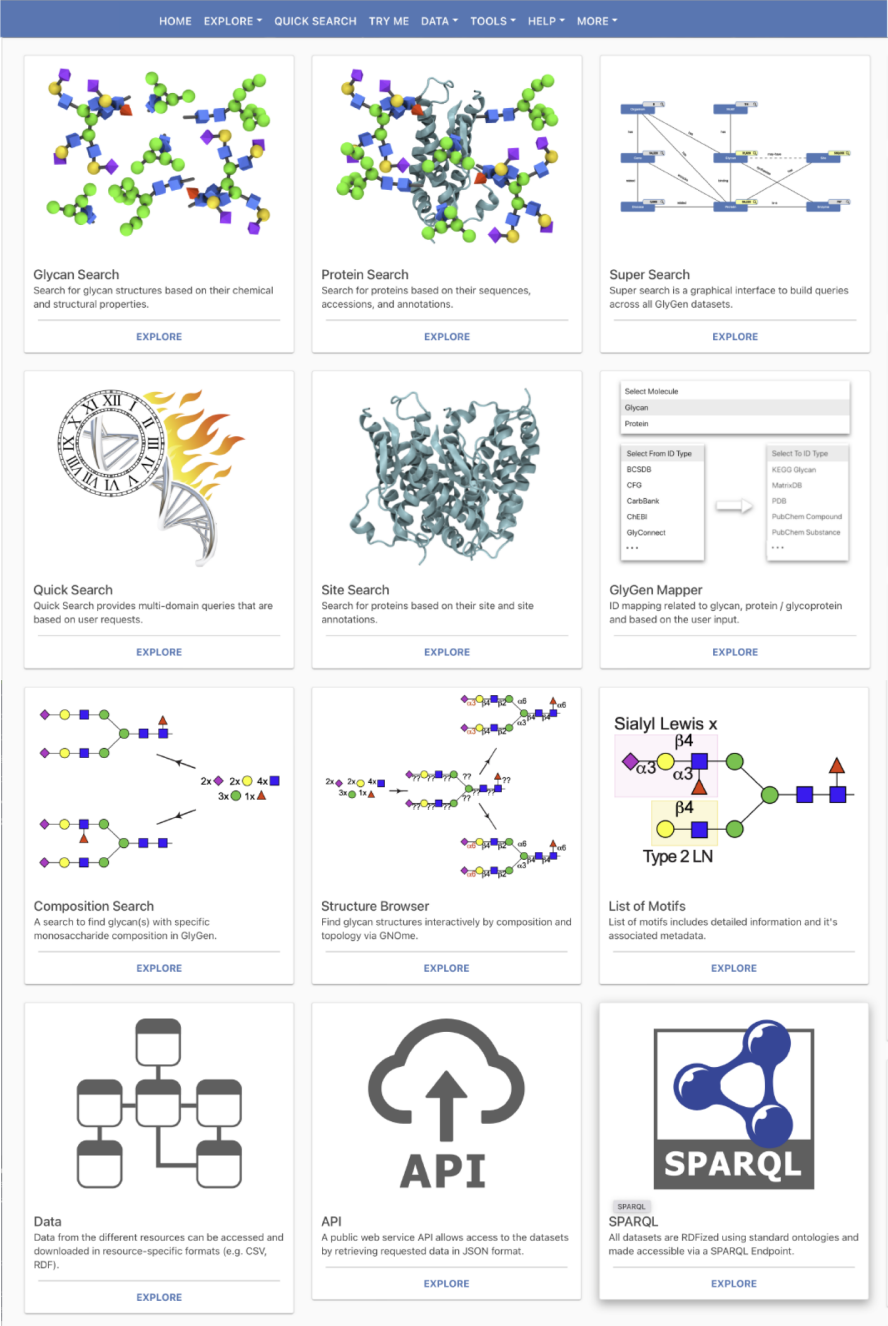
GlyGen home page provides users with search interfaces that access glycan, protein, glycosylation site, glycan motif, structural subsumption relationship, and glycan composition data. Access to data files, APIs, and a SPARQL end point are also provided for more advanced users.

GlyGen was initially developed by CCRC (University of Georgia, USA) and George Washington University collaborators with the support of the NIH Glycoscience Common Fund.^9^ It facilitates the exploration of data related to glycans, proteins, and glycoproteins in *Homo sapiens*, *Mus musculus*, *Rattus norvegicus*, Hepatitis C viruses, SARS-CoV-2, and SARS-related coronavirus. Data is accessible through an intuitive web portal (Figure 4; https://www.glygen.org) or as resource-specific (CSV, RDF) format files (https://data.glygen.org). Glygen also provides a number of tools to explore these data sets.

### Glycans

Glycan data can be searched using simple, advanced, composition, structure, and substructure searches, each providing different routes to search data at various levels of expertise or granularity. The results of any of these searches lead users to a glycan details page, which lists a variety of useful information, including GlyTouCan accession number, mass values, composition string and glycan type, organisms in which the glycan is found, any names given to the glycan, motif information, list of associated proteins, glycan binding proteins, enzymes involved in its biosynthesis, related glycans in terms of ambiguity (often used by mass spectrometrists when analyzing glycomics data), tissues and cell lines in which the glycan is expressed, and publication information.

### Glycoproteins

Similar to glycans, glycoproteins can also be searched for by various categories, including disease, gene, glycan, organism, pathway, or protein name/ID. The resulting protein details page has a plethora of information gathered from various resources including relevant cross-links; detailed glycan-related information is also presented, including site-specific glycan modification at the composition and structural level. This section also divides the information into those that have been reported in the literature (with and without glycan data), those that have been predicted, and those that have been mined from the literature. Phosphorylation and glycation information is also provided when available.

### Tools

The tools section of GlyGen provides a variety of ways to explore the data in GlyGen. ID mapping, glycan motifs, BLAST, Sand Box, and Structure Browser. These tools allow users to search the glycans and proteins in GlyGen based on different parameters. For example, glycans containing certain motifs such as sialyl-Lewis X can be searched via the glycan motifs, or glycans that are known to be involved in specific biosynthetic pathways can be searched using the Sand Box.

### Integration of Glycan and Glycosylation Data with Other Data Types

GlyGen contributes its data and its experience in the glycoinformatics domain to recently expanding efforts that are integrating large data sets funded by the NIH, such as GTEx, HuBMAP, HMP, LINCS, SPARC, IDG, Metabolomics Workbench, and others (https://commonfund.nih.gov/dataecosystem). Under the shared umbrella of the Common Fund Data Ecosystem (CFDE), these groups provide access to harmonized data sets that include gene expression, disease phenotypes, druggable targets, metabolomics, microbiome dynamics, cell signatures, anatomic correlates of disease processes, and others to allow investigators to ask cross-cutting questions in novel ways. A key component of this effort is the development and adoption of shared metadata models and well-developed ontologies to ensure that all data types are findable, accessible, interoperable, and reusable (FAIR). The CFDE data portal (https://nih-cfde.org) juxtaposes glycoscience data with other data types, increasing the likelihood that users currently unaware of the importance of glycans and glycosylation may find themselves exploring the resources provided by GlyGen and, through our interconnections, the complementary resources available through all GlySpace Alliance members.

## Summary

In summary, the members of the GlySpace Alliance have developed their respective resources from various perspectives, as described in more detail in ref (51). In short, Glyco@Expasy aims to provide data from the user’s perspective along the lines of Expasy’s approach, while GlyCosmos attempts to provide extensive coverage of glycan-related omics data, and GlyGen focuses on integration with genes, proteins, and other data types. Depending on the user, one portal may be easier to use or more appropriate for their research domain. The Alliance members meet periodically to discuss not only the sharing of their resources and data integrity but also the means to make their resources more easily accessible to users.

We make note that all GlySpace Alliance members are continuously working on improving their resources to adhere to the FAIR principles. All data is freely available under CC-BY-4.0 license. Users are free to share and adapt, transform, and build upon the material for any purpose, including commercially as long as they provide attribution. Moreover, all members of the Alliance use well-known standards and ontologies and provide APIs and SPARQL end points as additional access points, ensuring that the data is findable, accessible, interoperable, and reusable.^52^ The homepage at http://glyspace.org is updated to reflect this, and user feedback is always welcome to improve these resources.

As these resources continue to develop and expand the integration with other omics resources, we envision that users would be able to access the GlySpace Alliance homepage to easily navigate the plethora of glyco-related resources currently available. Whether the user is a student new to the field or a veteran in an orthogonal field to glycobiology, they should be able to easily find the data that they are targeting. This data should be annotated with provenance and references to the literature along with educational resources to learn more about the data of interest. Conversely, these data should become better integrated with major genomics, proteomics, lipidomics, and metabolomics databases, such that the importance of glycosylation can be understood from various contexts. In addition, model organism databases could have improved integrations with the functional aspects of glycosylation via the GlySpace Alliance. As a whole, we hope to introduce the concept of an ”Expanded Central Dogma” including glycosylation as a standard to enlighten the life sciences.
